# A case report of brainstem hemorrhage due to *Rhizopus delemar*-induced encephalitis diagnosed by metagenomic next-generation sequencing (mNGS)

**DOI:** 10.1186/s12879-023-08192-1

**Published:** 2023-04-17

**Authors:** Shuhua Xie, Zhaohui Lai, Han Xia, Mingze Tang, Jinxing Lai, Qing Liu, Zhijuan Lu, Dehai He, Jiangli Qi, Xianghong Liu

**Affiliations:** 1 Department of Neurology, Ganzhou People’s Hospital, Jiangxi, 341000 China; 2 Department of Scientific Affairs, Hugobiotech Co., Ltd, Beijing, 100176 China

**Keywords:** *Rhizopus delemar*, Encephalitis, mNGS, Infection, Clinical diagnosis

## Abstract

**Background:**

*Rhizopus delemar* is an invasive fungal pathogen that can cause fatal mucormycosis in immunodeficient individuals. Encephalitis caused by *R. delemar* is rare and difficult to diagnose early. Clinical detection methods for *R. delemar* include blood fungal culture, direct microscopic examination, and histopathological examination, but the detection is often inadequate for clinical diagnosis and can easily lead to missed diagnosis with delayed treatment.

**Case presentation:**

We report a case of a 47-year-old male with brainstem hemorrhage caused by encephalitis due to *R. delemar*. The patient had a history of hypertension, type 2 diabetes, and irregular medication. No pathogens were detected in cerebrospinal fluid (CSF) and nasopharyngeal secretion cultures. *R. delemar* was identified by metagenomic next-generation sequencing (mNGS) in CSF, and in combination with the patient’s clinical characteristics, encephalitis caused by *R. delemar* was diagnosed. Antibiotic treatment using amphotericin B liposome in combination with posaconazole was given immediately. However, due to progressive aggravation of the patient’s symptoms, he later died due to brainstem hemorrhage after giving up treatment.

**Conclusions:**

mNGS technique is a potential approach for the early diagnosis of infections, which can help clinicians provide appropriate antibiotic treatments, thus reducing the mortality and disability rate of patients.

## Background

*Rhizopus delemar* belongs to a subtype of molds under Mucoraceae, Mucorales, and is an opportunistic pathogen that lives in decaying vegetated soils. The main species that can cause mucormycosis include *Rhizopus*, *Mucor*, and *Absidia*. As a common cause of mucormycosis, *R. delemar* can involve any tissue and organ, causing disseminated disease with a high mortality rate of over 90% [1–3].

Mucormycosis, also known as zygomycosis, is a serious fungal infection that occurs mainly in immunosuppressed population, including patients with hematologic malignancies, organ transplant recipients, and diabetic patients with poor glycemic control [4]. The mucormycosis-related pathogens have the ability to invade blood vessels and can lead to vasculitis and vascular thrombosis, causing massive infarction and necrosis [5]. The signs and symptoms of mucormycosis are non-specific and require a combination of clinical manifestations, pathology, and laboratory diagnosis. Direct microscopic examination, fungal culture, and histopathology can be used for the diagnosis of mucormycosis, but the detection process is time-consuming and has low sensitivity [1], and the delay in diagnosis leads to untimely treatment of patients with mucormycosis and high mortality. It should be noted that not all mucormycosis cases are immunocompromised, and there has been a worrying increase in infection rate in immunocompetent people in recent years. Bala et al. [6] found that 24% of patients with mucormycosis had no risk factors.

With increased awareness of clinicians in diagnosis and treatment, improved diagnostic techniques, and an increase in susceptible populations, more and more mucormycosis cases are being diagnosed worldwide [7]. Here we report a case of brainstem hemorrhage due to encephalitis caused by *R. delemar*, which has been detected by mNGS using the cerebrospinal fluid (CSF).

## Case presentation

**Fig. 1:**
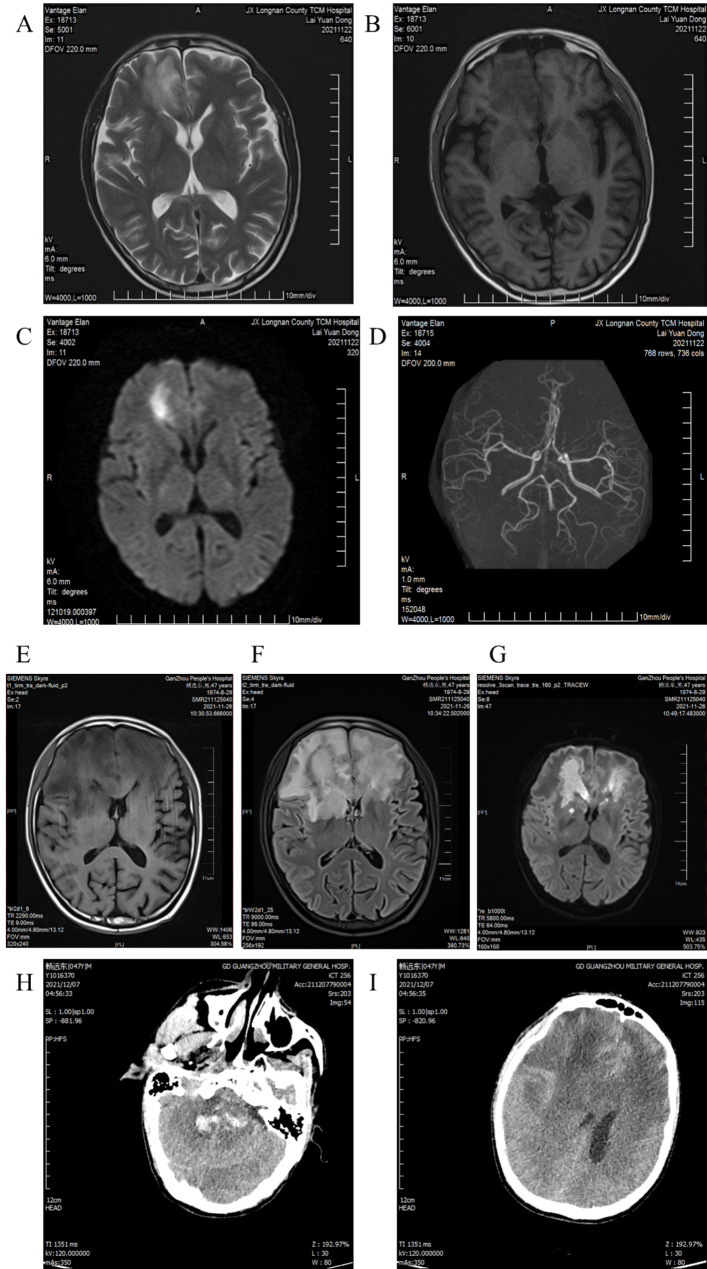
Cranial MRI and MRA images of the patient. A-D, Images acquired one week before admission. E-G, Images acquired on the second day of admission. H-I, Images acquired on the eighth day of admission

A 47-year-old male patient was admitted due to “repeated headache for 1 week, with convulsion and babbling for 1 day” on November 24th, 2021. He had a history of hypertension and diabetes mellitus and was on irregular insulin control with poor glycemic control; he was on intermittent benazepril for blood pressure control. He denies any history of head trauma. One week before admission, the patient developed headache without obvious inducement and paroxysmal dull pain in the forehead and was admitted to Longnan Hospital of Traditional Chinese Medicine. There were no abnormal symptoms of the patient, such as fever, cough or expectoration, nausea or vomiting, disturbance of consciousness, limb twitching, and babbling. RT-qPCR for SARS-CoV-2 was negative. The brain (MRI + MRA) examination was completed and revealed abnormal signal shadows in the right frontal lobe (Fig. 1A-D). The patient was diagnosed as “cerebral infarction without excluding encephalitis” in the local hospital, and was given “treatment to improve circulation, nourish nerves, antiviral therapy and other treatments”, but the results were poor.

On the way to our hospital for referral, the patient presented with a gibberish convulsion that manifested itself as unresponsiveness to calls, muscle rigidity in the extremities, upturned eyes, and closed teeth. On admission, physical examination revealed clear consciousness, clear speech, slightly unresponsive, cooperative to examination, decreased memory and comprehension, no ptosis, equal and round bilateral pupils, 3.0 mm in diameter, sensitive light reflexes, normal bilateral eyeball movements, symmetrical bilateral frontal lines, symmetrical bilateral nasolabial folds, centered tongue extension, presence of gag reflexes, no atrophy of limb muscles, grade 5 muscle strength of bilateral upper and lower limbs, normal muscle tone, symmetrical tendon reflexes of the extremities (+), negative Babinski sign, soft neck without resistance, negative Kernig sign, and negative Brudzinski’s sign. Preliminary diagnosis: (1) abnormal intracranial signal to be examined: intracranial tumor or viral encephalitis is suspected. (2) grade 2 hypertension with very high risk. (3) Type 2 diabetes with poor blood glucose control. Biochemistry: white blood cell count (WBC) 7.93 × 10^9/L (3.5–9.5), neutrophil percentage 83.20% (40–75), hemoglobin 94.00 g/L (130–175), sodium 126.46 mmol/L (137–147), chloride 88.40 mmol/L (99–110), four-item coagulation: PT: 22.60s (9–13); INR: 1.94 (0.8–1.5); APTT: 81.80s (20–40); AT-III: 73.30% (75–125); PT%: 32.70% (70–130). Urine test: glucose 2+.

On November 24th, 2021, the first lumbar puncture CSF test in our hospital showed intracranial pressure of 85 mmH_2_O, WBC 450 × 10^6^/L, neutrophil percentage 86.2%, lymphocyte percentage 13.8%, glucose 7.34 mmol/L (synchronous blood glucose 16.8 mmol/L), chloride 117.15 mmol/L, creatine kinase 2.1 U/L, and CSF protein quantification 81.60 mg/dl. In order to further detect the pathogen, the CSF was submitted for PACEseq mNGS (Hugo Biotech Co., Ltd.) on November 25th, 2021 (the second day of admission). Ceftriaxone (2 g Q12H) for anti-infection, acyclovir (0.5 g Q8H) for anti-virus, oxcarbazepine for anti-epilepsy, glucose-reducing therapy, maintenance of water-electrolyte balance and other symptomatic and supportive treatment were given.

**Fig. 2:**
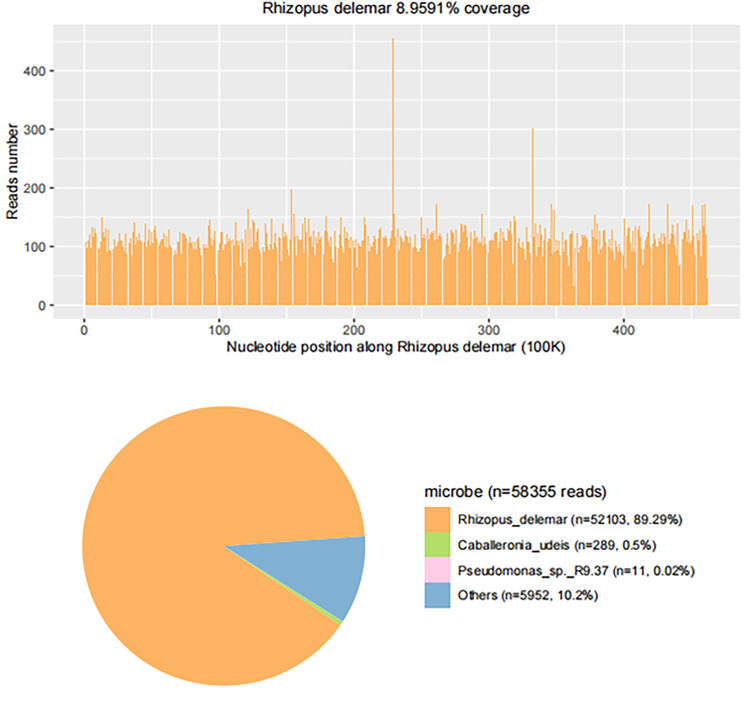
On the third day of admission, the CSF PACEseq mNGS showed 52,103 specific sequences with 8.9591% coverage

On the second day of admission, the patient began to develop fever, with a maximum temperature of 38.6 ℃, and the temperature fluctuated between 36.8 ℃ and 39 ℃ during hospitalization. Brain MRI scan with enhancement was completed and revealed “a wide range of abnormal signals in the bilateral frontal lobes, basal ganglia, right insula and corpus callosum, with partial leptomeningeal thickening and abnormal enhancement in the bilateral frontal lobes, suggesting the possibility of meningoencephalitis” (Fig. 1E-G). On the third day of admission, PACEseq mNGS of CSF showed 52,103 specific sequences of *R. delemar* with coverage of 8.9591% (Fig. 2). CSF bacterial and fungal culture and nasal secretion cultures showed no abnormalities; acyclovir was subsequently discontinued and switched to liposomal amphotericin B (10 mg qd) and oral posaconazole suspension (5 ml tid) for antifungal therapy.

On the third day of admission, CSF was reexamined: intracranial pressure 210 mmH_2_O, CSF protein quantification 65.76 mg/dl, WBC 57 × 10^6^ /L, and neutrophil percentage 46.3%. The patient’s mental state changed from awake to lethargy, which was considered to be caused by high intracranial pressure, and mannitol (125 ml q8h) was added to reduce the intracranial pressure. Blood biochemistry showed abnormal liver function and renal function electrolytes, manifested as ALT (alanine aminotransferase) 79.86 U/L, AST (aspartate aminotransferase) 111.13 U/L, albumin 28.74 g/L, creatinine 99.70 umol/L, and potassium 2.64 mmol/L. On the basis of the original antifungal therapy (liposomal amphotericin B adjusted to 20 mg qd) and other treatments, maintenance treatments such as liver protection and albumin supplementation were added, but the patient’s consciousness progressively worsened.

On the fourth day of admission, the patient began to be lethargic, and still had recurrent high fever without further convulsions. At the request of his family, he was transferred to the Chinese People’s Liberation Army General Hospital on the 7th day of admission, and on the 8th day of hospitalization, he suddenly developed accelerated heart rate, elevated blood pressure, and deep coma. Repeated brain CT revealed “brainstem hemorrhage and ruptured into the fourth ventricle; brain hernia” (Fig. 1H-I), and the family gave up treatment. Telephone follow-up revealed that the patient died after returning home.

## Discussion and conclusions

Fungal infections of the central nervous system (CNS) are known to have diverse clinical manifestations, mainly manifested as meningitis, encephalitis, hydrocephalus, brain abscess, and stroke syndrome [8]. Neurological disorders are the leading cause of disability and the second leading cause of death worldwide [9]. The common route of transmission of the pathogen is inhalation or inoculation due to trauma or surgery, followed by hematogenous or continuous spread. The diagnosis of fungal infections of the CNS is very difficult because their presentation is usually nonspecific. Rapid determination of the cause of infection and the application of precise treatment are essential to reduce mortality.

The annual incidence of mucormycosis is estimated to be 1.7 per million people [10]. The main route of infection with *R. delemar* is inhalation of sporangial spores, with pulmonary and nasal-brain infections being the most common [11]. Neutropenia or glucocorticoid therapy is considered a risk factor for rhino-cerebral and pulmonary infections, while diabetic patients are prone to have rhino-cerebral mucormycosis [12]. The largest group (58.9-86.7%) of patients with nasal-orbital-cerebral mucormycosis are diabetic patients, particularly patients with diabetic ketoacidosis [3]. There is also a case of basal ganglia mucormycosis, who did not have any known cause of immunosuppression, but with a history of drug injection [13]. Even with antifungal treatment and surgery, there is still a mortality rate of approximately 50-99% within a few months of diagnosis, depending on the accurate diagnosis and level of transmission at the time of treatment [14]. Mucormycosis has a marked tendency to invade blood vessels, resulting in thrombosis, necrosis, and tissue infarction, with a high mortality rate [15]. In this case, the patient had a history of diabetes but denied having any other cause of immunosuppression or history of IV drug abuse. The epidemiology should be further explored. The patient died after giving up treatment due to brainstem hemorrhage during treatment. Combined with imaging data, the patient was found to have new intracranial infarction and hemorrhagic foci, which was related to *Rhizopus* having unique vascular invasion ability and could lead to vasculitis and vascular thrombosis. Death from pulmonary venous thrombosis complicated by hemorrhagic infarction due to *R. delemar* invasion of blood vessels has been reported in patients with pulmonary mucormycosis who received heart transplants [16].

In the course of rhino-orbital-cerebral mucormycosis, spacy-occupying lesions in the maxillary sinus, ethmoid sinus, periorbital region, and intracranial space are often seen; the most common symptoms include headache, especially facial pain, periorbital edema, often accompanied by decreased visual acuity, fever, diplopia, rhinitis, and neurological function decline. Black nasal discharge, crusting, alar necrosis, ulceration, and palatal perforation are also common. Histopathological examination of tissue specimens (CSF, biopsy specimens, surgically resected specimens, and autopsy material) shows some hyphae. In addition, the middle nasal tract is prone to mucosal swelling due to narrowing and obstruction, sinus orifice obstruction, decreased mucosal transmission, reduced sinus cavity ventilation, and decreased pH, such conditions are conducive to fungal growth. Thus, when susceptible individuals inhale fungal spores, turbinate or alveolar infection usually occurs first [17]. For this patient, we contacted an otolaryngologist to assist in observing the patient’s bilateral nasal tract, and no obvious occupying lesions or necrosis or exudation were found, and nasopharyngeal swabs taken for culture also revealed negative results.

According to the EORTC/MSG criteria, the diagnosis of invasive fungal infections requires consideration of multiple complex factors, including host risk factors, pathological and mycological analyses, as well as related clinical signs and imaging features. CT and MRI images of fungal infections in different diseases can be the same; nonspecific focal lesions, edema, or hemorrhagic lesions can be seen in MRI images; therefore, CT and MRI techniques can only be used as adjuncts in the diagnosis of fungal infections of the CNS [18]. These imaging techniques can help with localization, but qualitative diagnosis still requires histological examination. In the intracranial imaging of this patient, the lesions involved bilateral frontal lobes, basal ganglia, insula and corpus callosum, and the meninges were thickened and enhanced; the images could only be preliminarily characterized as meningoencephalitis, but no specific pathogen could be identified.

Biopsy of lesioned tissue is an important tool for the diagnosis of invasive fungal diseases, and biopsy can obtain pathological specimens, including brain tissue and meninges [19], but biopsy of the CNS is riskier in critically ill patients, especially those with thrombocytopenia or neutropenia. CSF cytology has certain auxiliary value in the diagnosis of neurological infection, especially GMS and PAS staining, while the cytological type of CSF depends on the pathogen of infection [20]. Polymerase chain reaction (PCR) test can accurately diagnose pathogens, especially in patients whose clinical conditions do not allow invasive procedures [21], but the use of PCR-based tests still needs to be further standardized. In recent years, CSF mNGS technology has been gradually applied to the diagnosis of CNS infections. This technology allows for non-targeted detection of nucleic acids of pathogens such as bacteria, fungi, viruses and parasites present in clinical specimens. The results of a non-prospective study showed that CSF mNGS had a sensitivity of 73% and a specificity of 99% in the diagnosis of encephalitis and meningitis [22], which fully illustrated the utility of CSF mNGS in the diagnosis of pathogens in infectious diseases of CNS. In this case, we combined the patient’s risk factors (long-term history of diabetes), clinical signs (neurological manifestations, such as headache and limb twitching), and imaging features (invasive changes by cranial MRI) on admission, and tentatively diagnosed the patient possible fungal infection. CSF and nasopharyngeal secretion cultures were negative, and the family declined biopsy. Fortunately, the patient’s CSF mNGS test revealed 52,103 specific sequences of *R. delemar*, the patient was ultimately diagnosed with proven mucormycosis.

In conclusion, this paper reports a case of a patient with *R. delemar* detected by mNGS who died after symptomatic treatment and abandonment of treatment due to brainstem hemorrhage discharged from the hospital. Mucormycosis progresses rapidly after infection, often with vascular invasion and high mortality and disability rates, requiring early diagnosis and early treatment. Once a patient is suspected to have cerebral mucormycosis, CSF mNGS, PCR detection, routine blood and CSF culture, and histopathology are necessary for early and accurate diagnosis. Timely diagnosis and treatment combined with antifungal therapy are essential to reduce the mortality of cerebral mucormycosis.

## Data Availability

The datasets used and/or analyzed during the current study are available at https://ngdc.cncb.ac.cn/bioproject/browse/PRJCA012545.

## Abbreviations

mNGS: metagenomic next-generation sequencing
CSF: cerebrospinal fluid
MRI: magnetic resonance imaging
MRA: magnetic resonance angiography
WBC: white blood cell count
CNS: central nervous system
CT: computed tomography
PCR: polymerase chain reaction
